# *GmLac55* Enhanced Soybean Resistance Against Soybean Cyst Nematodes Through Lignin Biosynthesis

**DOI:** 10.3390/ijms26136304

**Published:** 2025-06-30

**Authors:** Hui Wang, Shumei Liu, Han Wang, Dige Luo, Chuanwen Yang, Songjie Qi, Min Wang, Yubo Jia, Yuxi Duan, Chen Liu, Qiumin Chen

**Affiliations:** 1 College of Bioscience and Biotechnology, Shenyang Agricultural University, Shenyang 110866, China; wanghui@syau.edu.cn (H.W.); 2024220212@stu.syau.edu.cn (S.L.); 18640526656@163.com (H.W.); 2024240409@stu.syau.edu.cn (D.L.); 18762911399@163.com (C.Y.); qiqiqi18642583182@163.com (S.Q.); 2024220188@stu.syau.edu.cn (M.W.); 2021170072@stu.syau.edu.cn (Y.J.); 2 Plant Protection College, Shenyang Agricultural University, Shenyang 110866, China; duanyx6407@163.com; 3 Key Laboratory of Potato Genetic Improvement and Germplasm Innovation in Shanxi Province, Shanxi Agricultural University, Taiyuan 030031, China

**Keywords:** soybean, SCN, *GmLac55*, lignin

## Abstract

Soybean cyst nematodes (SCNs) are a significant disease that causes yield loss and reducing seed quality in soybeans (*Glycine max*). Developing SCN-resistant soybean varieties can minimize the need for insecticide use and reduce yield loss. Cinnamate-4-hydroxylase (C4H) and laccase (Lac) are key enzymes in the lignin synthesis pathway. In this study, SCN stress significantly promoted lignin accumulation in soybean roots and upregulated the expression of lignin signaling pathway genes *GmC4H* (Glyma.02G236500), *GmLac55* (Glyma.13G076900), and *GmLac85* (Glyma.20G051900). Using *Agrobacterium rhizogenes*-mediated transformation, the pNI900 expression vector was introduced into the soybean cultivar Williams 82 to generate *GmLac55*-overexpressing plants. The overexpression of *GmLac55* enhanced soybean roots resistance to SCN and inhibited the further development of J2 larvae. Our study presents a strategy for increasing SCN resistance in soybean through *Agrobacterium*-mediated targeted mutagenesis of the *GmLac55* gene.

## 1. Introduction

Soybean cyst nematode (*Heterodera glycines* Ichinohe, SCN) is a highly contagious soil-borne pathogen with a rapid spread rate and broad infection range. It invades soybean plants through the roots, disrupting their normal growth and development. The SCN’s diverse pathotypes, long-lived dormant stages, and wide host range make its control challenging. SCN disease is prevalent across China and has long been a global issue, posing a devastating threat to soybean yields [1]. Farmers primarily rely on chemical pesticides and crop rotation to mitigate this threat. However, the prolonged use of chemical pesticides not only fosters drug resistance in target pests but also contaminates land and water resources, impacts the environment, and escalates environmental management costs. Additionally, the sequence of crop rotation can influence soybean yields [2]. Developing SCN-resistant soybean varieties can reduce the need for insecticides, enhance soybean yields, and alleviate concerns related to pesticide use. Transgenic breeding has brought about significant advancements in plant breeding, and the cultivation of genetically modified crops has notably boosted global agricultural productivity over the past two decades [3]. Genetically modified cultivars that express candidate resistance genes have become crucial for effective SCN management [4].

Lignin, a key component of the plant secondary cell wall, is crucial for stem strength and rigidity [5]. It fortifies plant cell walls, offering physical support and protection against pathogens [6]. Composed of p-hydroxyphenyl (H), guaiacyl (G), and syringyl (S) phenylpropanoid units, lignin is a complex racemic aromatic heteropolymer [7]. Lignin is crucial for the resistance of plants to abiotic stresses [8]. Under drought stress, reduced lignin deposition enhances the water transport and boosts the drought resistance in *Arabidopsis* [9]. Lignin also holds immense industrial value. Engineering C4H mutant plants can significantly increase their saccharification efficiency [10]. Lignin deposition during plant–pathogen interactions serves as a physical barrier against pathogen invasion [11,12]. Relevant studies have shown that the expression of lignin biosynthesis genes and lignin levels increases with pathogen infection [13,14,15], where lignin was also found to restrict pathogen movement within the plant and thereby enhance its disease resistance [16].

The synthesis of lignin involves several key enzymes, including phenylalanine ammonia-lyase (PAL), cinnamic acid 4-hydroxylase (C4H), laccase (Lac), and 4-coumarate CoA ligase (4CL) [17,18]. Laccase, the terminal enzyme in the lignin synthesis pathway, is vital for lignin production [19,20]. Lignin, as a metabolite, serves as the first line of defense against nematode attacks by increasing the cell wall thickness through lignin deposition [21]. After lignin monomers are synthesized, they undergo dehydrogenation polymerization to form lignin [22]. Laccase’s primary function in plants is its involvement in lignification [23,24]. The current research largely focuses on microbial laccases, with less attention being paid to plant laccases and no reports existing on their role in soybean resistance to nematode infection. Laccase is capable of degrading lignin, promoting its polymerization, and facilitating lignin deposition [25,26,27]. By 2019, 93 *GmLac* genes had been identified in the soybean genome [22]. Ithal et al. identified 429 genes that are involved in lignin biosynthesis, stress, and defense responses and which were differentially expressed in the root tissues of 35,611 soybean transcripts before and after SCN infection [28]. Additionally, laccase genes are indispensable in plant responses to various abiotic and biotic stresses [29,30,31,32].

Soybean (*Glycine max*) is an important industrial crop that provides edible oil, vegetable protein, and active compounds. The precise mechanisms by which soybeans resist invasion and colonization by SCNs remain unclear. Studies have consistently highlighted the cell wall’s role as a physical barrier against various stresses [11]. Lignin, a crucial component of the plant secondary cell wall, has been shown to thicken cell walls and enhance the stress resistance [33,34]. Cinnamic acid 4-hydroxylase (C4H) and laccase, key enzymes in the lignin synthesis pathway, likely play indispensable roles in SCN resistance by regulating lignin production. Lignin and its metabolic processes are significantly involved in the resistance response of SCNs, exerting notable interference in and control over SCN disease. Therefore, investigating the effect of lignin synthesis genes that are involved in the resistance of soybean to SCNs will help elucidate the mechanisms by which lignin contributes to soybean resistance against SCNs.

In this study, we found that the lignin content in the roots of the susceptible variety Williams 82 and the resistant variety Xiaoliheidou (ZDD1412) showed significant differences after SCN inoculation. Simultaneously, the expression of the key lignin synthesis genes *GmCH4* and *GmLacs*, particularly *GmLac55*, changed significantly under SCN stress. Furthermore, an *Agrobacterium rhizogenes*-mediated genetic transformation system was employed for the overexpression of *GmLac55*, and the resistance to SCNs of *GmLac55-OE* plants was identified. The results showed that the overexpression of *GmLac55* had a significant effect of reducing nematode numbers at various stages and enhancing the resistance of soybean to SCNs. Findings from this study present a strategy for increasing the SCN resistance in soybean through targeted mutagenesis of the *GmLac55* gene.

## 2. Results

### 2.1. Lignin Response in Roots of SCN-Resistant and SCN-Susceptible Soybean Varieties

**Figure 1:**
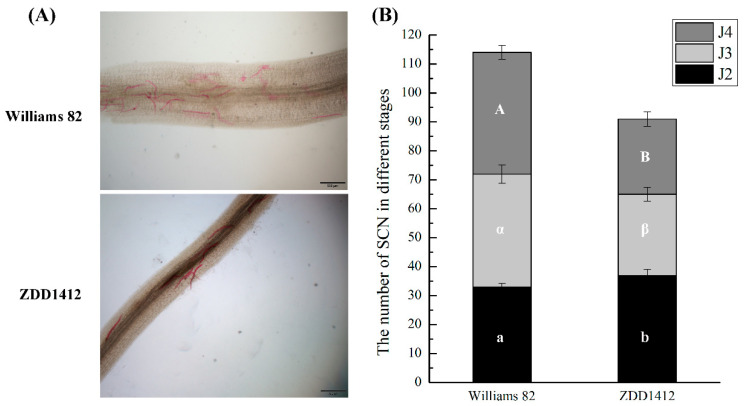
Soybean cyst nematode (SCN) in soybean roots. (**A**) Microscopic observation of SCN enrichment staining in root systems of different resistant soybean varieties. Scale bars: 500 μm. (**B**) Number of SCNs at different developmental stages in soybean roots. Different letters indicate significant differences: a or b for J2, α or β for J3, A or B for J4.

To investigate the mechanisms underlying the resistance of soybean to SCNs, we selected two soybean materials with differing resistance levels: the susceptible variety Williams 82 and the resistant variety ZDD1412. After inoculation with SCNs, acid fuchsin staining experiments revealed a higher number of infecting nematodes on the roots of the Williams 82 plants compared to the ZDD1412 plants, indicating that ZDD1412 exhibits significant resistance to SCN infection (Figure 1A). The SCN counts in the soybean roots revealed that, in the Williams 82 groups, the total number of SCNs was higher than that in the ZDD1412 groups (Figure 1B). When comparing the number of SCNs at the same nematode developmental stage between the Williams 82 groups and the ZDD1412 control, significant statistical differences were found in the number of J2s, J3s, and J4s. Specifically, the proportion of J2s was significantly higher in the ZDD1412 groups, while the proportions of J3s and J4s were significantly higher in the Williams 82 groups (Figure 1B). This indicates that ZDD1412 exhibits stronger resistance to SCNs, which is characterized by fewer accumulated SCNs in the root system and inhibited SCN development.

**Figure 2:**
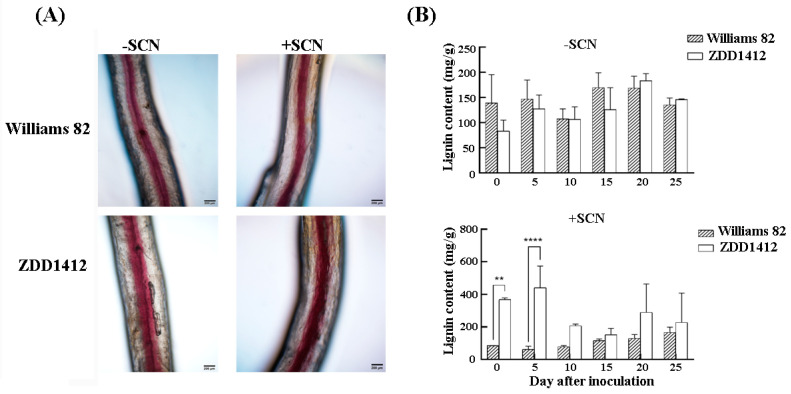
Effects of SCN inoculation on lignin content. (**A**) Phenotypic comparison of lignin staining in soybean roots before and after SCN inoculation (day 10). Scale bars: 200 μm. (**B**) Measurement of lignin content in soybean roots before and after SCN inoculation. Asterisks denote statistical significance: ** *p* < 0.01, **** *p* < 0.0001. Data represent three biological replicates.

The root cell wall serves as the first physical barrier against SCN invasion, with lignin being a crucial component of the cell wall. The lignin staining of SCN-infected soybean roots showed that, 10 days post-inoculation, the ZDD1412 roots had a substantial accumulation of lignin, whereas the lignin content in the Williams 82 roots remained largely unchanged before and after SCN inoculation (Figure 2A). Additional measurements of the lignin content across different lines revealed no significant difference between the Williams 82 and ZDD1412 plants under non-inoculated conditions. However, following SCN inoculation, the lignin content in the ZDD1412 roots was significantly higher than that in the Williams 82 (Figure 2B) roots. The results suggest that, under SCN stress, the ZDD1412 variety can enhance lignin synthesis to combat the stress, whereas the Williams 82 variety can only synthesize a small amount of lignin, showing greater sensitivity to SCN.

### 2.2. Lignin Signaling Pathway Regulates Soybean Resistance to SCN

**Figure 3:**
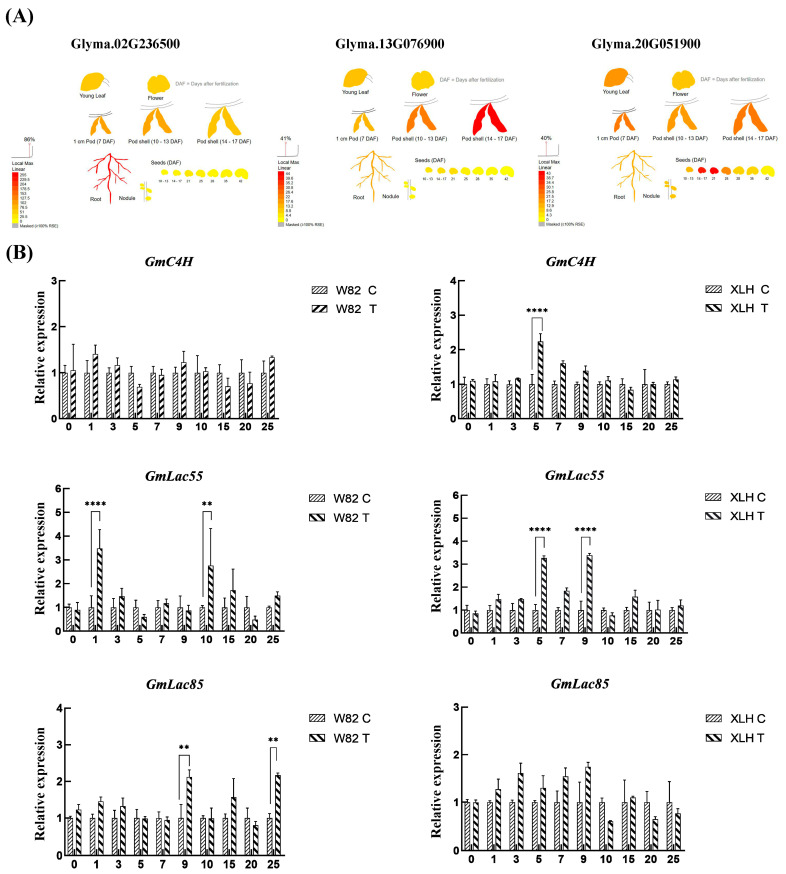
Expression changes oflignin signaling genes under SCN stress. (**A**) Predicted gene expression of lignin signaling genes *GmC4H* (Glyma.02G236500), *GmLac55* (Glyma.13G076900), and *GmLac85* (Glyma.20G051900). (**B**) Relative expression levels of *GmC4H*, *GmLac55*, and *GmLac85* in roots at various day post-SCN inoculation. Asterisks indicate statistical significance: ** *p* < 0.01, **** *p* < 0.0001. Data represent three biological replicates.

To identify the key factors involved in the SCN resistance response in resistant materials, we further examined the expression patterns of the keygenes involved in lignin signaling, namely *GmC4H* (Glyma.02G236500), *GmLac55* (Glyma.13G076900), and *GmLac85* (Glyma.20G051900) under SCN stress. To predict the expression levels of *GmC4H*, *GmLac55*, and *GmLac85* in different tissues, we utilized the online resource available at http://bar.utoronto.ca/ (accessed on 22 May 2025) (Figure 3A). *GmC4H* is predominantly expressed in the primary root of soybean, and its expression level remains largely unchanged, or even shows no significant variation, before and after pathogen infection. In contrast, *GmLac55* and *GmLac85* are highly expressed in the root tips of soybean, and their expression levels are significantly upregulated both before and after infection. This suggests that, compared to *GmC4H*, *GmLac55* and *GmLac85* may play more crucial roles in the plant’s response to stress.

*GmC4H*, *GmLac55*, and *GmLac85* were induced in both susceptible and resistant soybean varieties after SCN inoculation (Figure 3B). In the roots of the Williams 82 variety, the expression of *GmC4H* showed little difference compared to the control. The expression of *GmLac55* was significantly higher than the control 1 day after inoculation (dpi) and 10 dpi, reaching 3.27 and 3.39 times that of the control, respectively. The expression of *GmLac85* peaked at 9 dpi, at 2.12 times that of the control (Figure 3B). In ZDD1412 roots, the expression of *GmC4H* peaked at 5 dpi, at 2.24 times that of the control. The expression of *GmLac55* was significantly higher than the control on 5 dpi and day 9 dpi, reaching 3.27 and 3.39 times that of the control, respectively. Although the expression of *GmLac85* was upregulated, the difference was not statistically significant compared to the control (Figure 3B). Overall, after SCN inoculation, the expressions of *GmC4H*, *GmLac55*, and *GmLac85* increased at different time points in the ZDD1412 variety. It appears that SCNs partially induce the expression of *GmC4H* and strongly induce the expression of *GmLac55* and *GmLac85*. Among these, *GmLac55* shows the most significant expression under SCN stress in the ZDD1412 variety, coinciding with the SCN colonization stage, and may play an important role in restricting SCN colonization and further development within the soybean plant.

### 2.3. Overexpression of GmLac55 Enhances Soybean Roots Resistance to H. glycines

**Figure 4:**
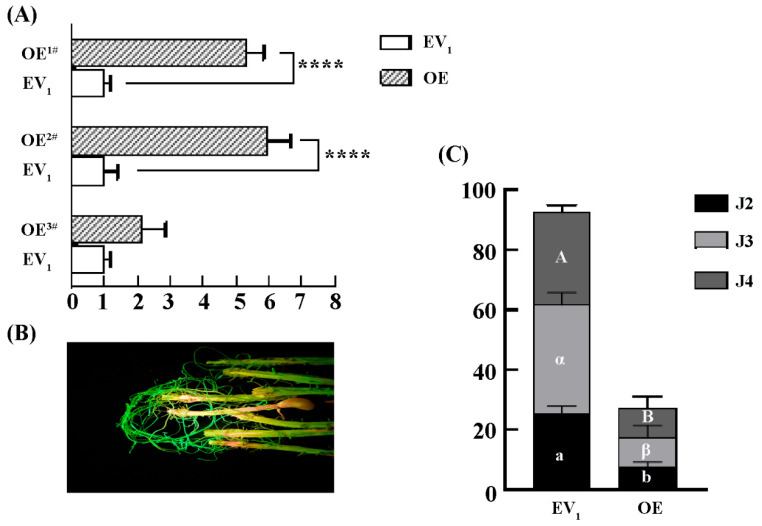
Overexpression of *GmLac55* enhances soybean root resistance to SCNs. (**A**) Relative expression levels of *GmLac55* in overexpressed groups (OE, n = 5) and empty vector control groups (EV_1_, n = 3). Asterisks indicate statistical significance: **** *p* < 0.0001. (**B**) Fluorescent hairy roots (OE) can be easily distinguished using the LUYOR-3415RG as the excitation light source. (**C**) Total number of SCNs in soybean roots and demographic assays of SCNs at different stages between overexpressed groups (OE, n = 5) and empty vector control groups (EV_1_, n = 5) (one-way ANOVA; in EV_1_ versus OE, a or b indicates significant differences for J2, α or β for J3, A or B for J4).

We further investigated the function of *GmLac55* in the regulation of the resistance of soybean roots to SCNs. The overexpression of *GmLac55* was achieved through the induction of hairy roots using *Agrobacterium rhizogenes*. qPCR was employed to detect the expression level of *GmLac55* in hairy roots. In the overexpression (OE) groups (OE^1#^, OE^2#^, and OE^3#^), the relative expression level of *GmLac55* was five- and six-fold higher than that of the empty vector (EV_1_) control (Figure 4A). Moreover, laser detection of the GFP fluorescence confirmed the successful transformation of the cultured soybean hairy roots, as evidenced by the distinct presence of positive GFP signals (Figure 4B).

The transformed hairy roots were inoculated with SCNs and, at 12 dpi, the roots were stained using the acid fuchsin method. A significant statistical difference was observed between the OE groups and the EV_1_ control in terms of the total number of nematodes. In the OE groups, the total number of SCNs was lower than that in the EV_1_ control (Figure 4C). When comparing the number of SCNs at the same developmental stage between the OE groups and the EV_1_ control, significant statistical differences were found in the number of J2s, J3s, and J4s (Figure 4C). Additionally, the proportion of each SCN instar in the control group was J2/J3/J4 = 1:1.41:2.11, whereas, in the OE lines, it was J2/J3/J4 = 1:1.26:1.26. Based on these proportions, we concluded that, compared to the resistant variety, the development and transformation ratio from J2 to J3 and J4 was reduced in the OE lines. These results suggest that *GmLac55* may play an important role in limiting SCN invasion and its development in soybean roots.

## 3. Discussion

During plant–pathogen interactions, lignin deposition acts as a physical barrier to prevent pathogen invasion [11,12]. It has been demonstrated that the expression of genes involved in lignin biosynthesis and the levels of lignin itself rise in response to pathogen infection [13,14]. In our study, we assessed the lignin content in untreated plants and observed that the resistant variety exhibited higher lignin contents compared to the susceptible variety, which likely contributes to its enhanced disease resistance. Following SCN infection, the lignin content notably increased, especially on the fifth day in the resistant variety ZDD1412 relative to the control group, which aligns with prior findings [35]. Additionally, the expression of lignin biosynthesis-related genes, including *GmC4H*, *GmLac55*, and *GmLac58*, was significantly upregulated under SCN infection conditions. These results underscore the pivotal role of lignin in conferring soybean resistance to SCNs.

The cell wall, serving as a physical barrier that maintains the structure of plant cells, can prevent the invasion of pathogens such as fungi, bacteria, and nematodes [36]. Lignin, as a key component of the cell wall, endodermis, and casparian strip, plays a crucial role in the stress regulation and cell wall resistance of plants [37]. Focusing on how lignin affects the cell wall and subsequently influences the resistance of soybean to SCNs, we detected significant changes in the expression levels of the lignin biosynthesis genes *GmC4H*, *GmLac55*, and *GmLac85* under SCN stress. Lignin is deposited during the interaction between plants and pathogens and is considered a physical barrier against pathogen invasion [11,12]. We further generated soybean hairy roots that overexpress *GmLac55*. Under SCN stress, Lac55 overexpression significantly enhanced the resistance of soybean to SCNs. This study lays the foundation for the development of disease-resistant plant varieties, which may rely on the accumulation of lignin.

Lignin, a vital component of plant cells, significantly influences the growth, infection, and development of SCNs. In the face of various stresses, lignin collaborates with other signaling molecule-mediated resistance pathways, which indicates its active participation in plant defense responses. This highlights the significant role of lignin in enhancing plant disease resistance. The SCNs significantly impact the yield and quality of soybean. Traditional pest control methods are often labor-intensive and time-consuming. Transgenic breeding has emerged as a crucial breakthrough in developing insect-resistant crops. Here, we present a strategy to enhance the SCN resistance in soybean through *Agrobacterium rhizogenes*-mediated targeted mutagenesis of the *GmLac55* gene. This approach can expedite the breeding process for insect resistance while minimizing potential health concerns for humans. Genetic transformation studies in soybeans further demonstrate that the overexpression of *GmLac55*, linked to the lignin synthesis pathway, can impact SCN infection and development. However, the regulation of *GmLac55* by salicylic acid (SA) and jasmonic acid (JA) signaling pathways remains a critical area that requires further investigation. For instance, the synergistic action of *GmLac55* with other resistance genes could enhance the overall defense response, potentially through the reinforcement of cell wall structures or the activation of additional defense pathways. Conversely, *GmLac55* may also exert independent effects, contributing to resistance through unique mechanisms such as the modulation of oxidative stress responses. Moreover, the functions of other *GmLac* genes in the resistance of soybean to SCNs are also key areas that need to be uncovered in the future.

## 4. Materials and Methods

### 4.1. Plant Materials and Growth Conditions

The susceptible variety Williams 82 and the resistant variety Xiaoliheidou (ZDD1412) were employed in this experiment. Following chlorine disinfection and bud acceleration, the seeds were cultured in an artificial climate chamber at 25–28 °C with a 12 h light and 12 h dark cycle. Williams 82 was used for genetic transformation. After the transformation procedure, the transformed plants were cultured for 14–21 days under the same conditions of 25–28 °C, 12 h of light, and 12 h of darkness.

### 4.2. The Acquisition of Nematodes

Soybean cyst nematode (*Heterodera glycines* Ichnohe, SCN) race 3, one of the most widely distributed races in China, was tested in this study. SCN-infested soil was mixed 1:1 with sterilized fine sand. Soybean seeds were sown to make SCNs grow and multiply. The cultivation lasted for two months, SCNs were separated from the infested soil, and the eggs were collected and incubated in Baermann Funnels at 27 °C, avoiding light. The hatched second-stage juveniles (J2s) were collected daily and new distilled water was added. In order to ensure the activity of J2s, the collection process was completed within one week. The isolated J2 SCNs were utilized to infect various soybean materials.

### 4.3. Lignin Dyeing and Content Determination

Lignin changes before and after inoculation were assessed via phloroglucinol staining. On the ninth day post-nematode inoculation, secondary roots with similar morphology were harvested and rinsed with tap water. The roots were then sectioned with a blade and promptly submerged in a fixative solution (95% ethanol: glacial acetic acid (*v*/*v*) = 1:1) for 24 h. Following this, the roots were rinsed with distilled water and subsequently immersed in a saturated chloral hydrate solution (100 g chloral hydrate dissolved in 50 mL ddH_2_O). Vacuum treatment was applied for 10 min using a vacuum pump, after which the samples were kept at room temperature until they became transparent. The roots were then soaked in a 1% phloroglucinol solution (5 g phloroglucinol, 25 mL 95% ethanol, and ddH_2_O to a final volume of 500 mL) for 5 min, followed by the addition of a few drops of concentrated hydrochloric acid. The samples were observed and photographed under a microscope. Lignin content was determined using a kit (BC4200) from Beijing Solarbio Company (Beijing, China).

### 4.4. RNA Isolation and Real-Time Quantitative PCR Analysis

Total RNA was extracted from soybean roots using TRIzol reagent (Beijing Kangwei Century Biotechnology, CW0581W, Beijing, China). RNA concentration was measured using a NanoVue spectrophotometer (Monad, Wuhan, China) for subsequent qRT-PCR and RNA sequencing (RNA-seq) analyses. For qRT-PCR, 1 μg of total RNA per sample was reverse transcribed into cDNA using the HiScript II Q RT SuperMix for qPCR (Vazyme, Nanjing, China) according to the manufacturer’s instructions. The qRT-PCR reactions were prepared in a total volume of 10 μL, containing 0.2 μM of each forward and reverse primer, 5 μL of SYBR Premix, 3.6 μL of ddH_2_O, and 1 μL of the cDNA template. The reactions were performed in an Industrial Diagnostic CFX96 Deep Well Real-Time PCR Instrument (Bio-Rad, Hercules, CA, USA) under the following conditions: initial denaturation at 95 °C for 30 s, followed by 40 cycles of 95 °C for 10 s, 60 °C for 30 s, and 95 °C for 15 s with simultaneous fluorescence measurement. A melting curve analysis was conducted at 60 °C for 60 s followed by 65 °C for 15 s. Three biological replicates were analyzed for each sample. Target gene expression levels were quantified using the 2^−ΔΔCt^ method [38], with untreated samples normalized to a relative value of 1. *Tubulin* (Glyma. 15G132200) was used as an internal reference gene. All primers used for qRT-PCR are listed in Table S1.

### 4.5. Vector Constructs and Plant Transformation

Total RNA extracted from soybean roots was reverse-transcribed to synthesize cDNA. The PCR-amplified fragments were ligated into the pNI9000 vector using the In-Fusion HD Cloning Plus kit (Takara, San Jose, CA, USA). The constructs were transformed into *Agrobacterium rhizogenes* K599 Chemically Competent Cell (Protein Interaction, Wuhan, China), with the pNI9000 empty plasmid serving as a control. The 5-day-old soybean seedlings were obliquely cut off near the hypocotyl, a drop of bacterial mass was applied to the incision site, and the seedlings were transplanted on wet vermiculite [39,40]. Fluorescent hairy roots can be grown after 30 days of culture. The positive hairy roots were checked by a LUYOR-3415RG Hand-Held Lamp (Luyor, Shanghai, China). In addition, qRT-PCR was used to detect the expression of the target gene. The primers used were listed in Table S1.

### 4.6. Identification of SCN Resistance of Overexpressed Plants

When the number of successfully transformed roots exceeds four, these roots can be utilized for subsequent nematode inoculation. If the number of transformed roots is insufficient, the soybean plants may not grow normally due to the reduced root mass after the primary roots are removed. For the transformed plants, only the transformed roots were retained, while the non-transformed roots were carefully excised. SCN J2 larvae, sourced from utricular soil, were mixed with 0.2% agar and evenly applied to the hairy roots. Twelve days post-inoculation, root staining was conducted using the sodium hypochlorite acid fuchsin method [41], and the number of nematodes at each developmental stage was meticulously counted.

### 4.7. Statistical Analysis

Graphpad Prism 9.0 and Microsoft Excel 2010 were used for data statistics and graph analysis. Comparisons between two groups were conducted using Student’s *t*-test. Significance levels are denoted as follows: * *p* < 0.05, ** *p* < 0.01, *** *p* < 0.001, and **** *p* < 0.0001. Comparisons among multiple groups were performed using two-way ANOVA, with *p* < 0.05 considered significant. All values are presented as means ± standard deviation (SD) from at least three biological replicates.

## 5. Conclusions

Our research indicates that the resistant soybean variety ZDD1412 exhibits slightly higher lignin content compared to susceptible varieties, with minimal SCN infection. Under SCN stress, the resistant variety ZDD1412 shows the significant upregulation of key lignin biosynthesis genes, particularly the *GmLac55* gene. This suggests that the resistance of ZDD1412 is directly related to the lignin signaling pathway. Expression analysis revealed that *GmLac55* is predominantly expressed in soybean roots and is upregulated in response to SCN stress, which indicates its involvement in the soybean root response to SCNs. Genetic transformation studies in soybeans further demonstrated that the overexpression of *GmLac55* can inhibit SCN infection and development. These discoveries shed light on the complex and diverse roles of lignin in SCN resistance mechanisms in soybeans. Future research should delve into the interplay between lignin, its signaling pathways, and other metabolic processes to uncover networks that directly or indirectly influence the development of SCNs.

## Data Availability

Data will be made available on request.

## Supplementary Materials

The supporting information can be downloaded at: https://www.mdpi.com/article/10.3390/ijms26136304/s1.
